# Regional Differences in Hepatocellular Carcinoma and its Surgical Treatment

**DOI:** 10.1155/1991/98621

**Published:** 1991

**Authors:** T. Ezaki, G. P. Stansby, K. E. F. Hobbs

**Affiliations:** 1 Department of Gastroenterological Surgery National Kyushu Cancer Centre Notame Minami-ku Fukuoka 815 Japan; 2 Hepato-biliary and Liver Transplantation Unit Royal Free Hospital School of Medicine Pond Street London NW3 2QG UK


*HPB Surgery*, 1991, Vol. 4, pp. 121–128
Reprints available directly from the publisher
Photocopying permitted by license only

© 1991 Harwood Academic Publishers GmbH
Printed in the United Kingdom

### REGIONAL DIFFERENCES IN HEPATOCELLULAR CARCINOMA AND ITS SURGICAL TREATMENT

T. EZAKI
*Department of Gastroenterological Surgery, National Kyushu Cancer Centre, Notame, Minami-ku, Fukuoka 815, Japan*

G.P. STANSBY, and K.E.F. HOBBS
*Hepato-biliary and Liver Transplantation Unit, Royal Free Hospital School of Medicine, Pond Street, London NW3 2QG, UK*

*(Received 13 December 1990)*

KEY WORDS: Hepatocellular carcinoma, hepatic surgery, hepatitis B virus

### INTRODUCTION

Hepatocellular carcinoma (HCC) is probably the commonest malignant tumour in males in the world and is responsible for approximately 1,000,000 deaths annually[1,2]. In China, most of Southeast Asia and Sub-Saharan Africa it occurs at a rate of 20 to 150 per 100,000 a year, whilst in the USA and Europe the rate ranges from 1 to 5 per 100,000 a year[3,4]. In all countries it shows a marked male preponderance.

### AETIOLOGY

Two major aetiological factors for HCC have been identified: Persistent Hepatitis B Virus (HBV) infection and liver cirrhosis. Both of these show marked geographical variations.

Evidence for the role of the HBV comes mainly from epidemiological studies. The worldwide prevalence of HBV infection is at least ten-times higher in Southeast Asia and Africa than in North America and Western Europe[5] and this parallels the incidence of HCC. In addition there seems to be an increase in the prevalence of HBV-markers in patients with HCC when compared to the local population. A prospective study in Taiwan determined that the relative risk of developing HCC was more than 200-fold greater in individuals who were HBsAg (Hepatitis B surface antigen) carriers[6] and the risk is said to be greater when the carrier state is acquired in early life[5,6,7]. The precise mechanism by which persistent HBV infection and the development of HCC are related remains largely unknown.

Address correspondence to: Mr. G.P. Stansby, Hepato-biliary and Liver Transplantation Unit, Royal Free Hospital,School of Medicine, Pond St., London, NW3 2QG, UK

Chronic carriage and cirrhosis seem to need to exist for 10–40 years before the tumour develops and only a fraction of persistent HBV carriers will eventually develop HCC[8]. It may be that HCC is initiated simply as a result of the increased cell turnover that occurs in cirrhosis or it may be that the HBV has a special role in initiating the tumour[1,5,9]. Integrated viral DNA has been demonstrated in both normal liver from chronic HBV carriers and in tumour tissue from such patients who have developed HCC[10]. More interestingly it has also been demonstrated in tumour tissue from patients who were negative for all HBV markers[11] suggesting that the role of the HBV in causing this tumour may be even more extensive than has been demonstrated by serological surveys. Whether other hepatitis viruses also play a role in causing HCC is currently unknown although evidence is emerging to suggest a role for Hepatitis C virus[12].

The other major risk factor identified is liver cirrhosis. The tumour seems to develop in cirrhotic livers about three times as often as in non-cirrhotic livers[13] and cirrhosis co-exists with HCC in approximately 70% of cases in both high and low risk countries[14–18]. In high-risk countries, cirrhosis and HCC usually share the common aetiology of HBV infection whilst in low-risk countries most cirrhosis is alcoholic in origin. There is no evidence that alcohol has a direct carcinogenic effect[4,19] and indeed the risk of HCC is increased in all types of cirrhosis. It may simply be that increased cell turnover increases the chance of malignancy or that the presence of cirrhosis in some way increase an individuals' susceptibility to environmental carcinogens. It has been stated that the risk is greater in macronodular cirrhosis irrespective of its aetiology, and that alcoholic cirrhosis and haemochromatosis carry particularly high risks[19]. However, if adjustment is made for male:female differences the risks may in fact be similar for all types of cirrhosis[20] including that secondary to chronic HBV infection.

Although over 90% of HCC can be correlated with HBV, or cirrhosis, or both, other minor risk factors also probably exist. In particular aflatoxin, a mycotoxin from the mould *Aspergillus flavus*, may be an important initiator in certain parts of Africa, perhaps by acting as a cocarcinogen with Hepatitis B. It is certainly a potent animal carcinogen and there is a close correlation between geographical HCC incidence and calculated aflatoxin exposure distribution[21]. Membranous obstruction of the inferior vena cava is a well recognised problem in the Far East, India and Southern Africa and it too has been suggested as being an aetiological factor for HCC. In one series from South Africa 48% had associated HCC[22]. Smoking, irradiation, cytotoxic therapy and sex hormone therapy may also have a role to play in occasional tumours. In addition there are reports that chronic infections of the liver such as schistosomiasis[23] and clonorchiasis[24] may be associated with HCC but the evidence here seems less strong.

### PATHOLOGY AND NATURAL HISTORY

It is often stated that small and encapsulated tumours are common in the East but rare in the West[25]. When present encapsulation seems to be a favourable prognostic sign[26–28] and this has been used by some to explain the better surgical results from Japan. However, the evidence is misleading. Most Western series are of surgically treated cases and depending on local referral practice a large number of patients may have been excluded. In addition there is no universally accepted definition of encapsulation and its reported incidence varies widely not only between countries but also between different centres in the same country. The Liver Cancer Study Group of Japan[26] reported macroscopic evidence of encapsulation in 80% of cases of HCC but Nakashima *et al.*[29] also from Japan, reported encapsulation in only 23% of their cases. Neither report defined how encapsulation was assessed. Nagorney *et al.*[30] from the USA showed tumour encapsulation in 20% of 110 cases and Kemeny *et al.*[31] in reporting 26 resected HCC's in cirrhotic patients in the West showed that histologically and morphologically they were similar to those encountered in the East. Small and encapsulated HCC is now being diagnosed and treated in the West and will probably be increasingly reported as it is increasingly looked for and as tumours are detected earlier.

Hepatocellular carcinoma is usually regarded as rapidly growing, but may not always be so. There seems to be a wide range of growth rates with some tumours growing rapidly and some slowly[32]. In some cases the disease may have been present for 3 years or more before detection[33]. There have only been two large studies where untreated small hepatocellular cancers were followed using ultrasound, one from Japan[34] and one from Taiwan[33]. Sheu *et al.* showed a mean doubling time of 117 days with a range of 29–398 days and found that doubling time was independent of the patient's age, sex, HBsAg status, degree of cirrhosis and histological type or grade. Nakashima *et al.*[29,35] showed that many small liver cancers are found at autopsy in cases of advanced cirrhosis. These may be slow growing or latent carcinomas which would never need surgery. The natural history of untreated small liver cancer less than 3 cm has been reported as showing a survival rate of 12.8% at three years after detection and any therapeutic modality must thus be judged against this figure[34]. Similar studies on growth rates in untreated cases are not available from the West and are unlikely ever to be performed. Unfortunately this makes it difficult to directly compare the natural history of Western and Eastern tumours. All that can be said is that there is currently no convincing evidence that HCC's from the East are fundamentally different in their growth rates to those from the West.

Fibrolamellar carcinoma, a variant of HCC, also demonstrates marked regional differences. Its distribution seems to be the reverse of the usual form and whilst it is rare in the East[35] it was found in 7% of one Western series[36]. A review of the literature found 143 reported cases which included only 3 from Asia[35] and the Liver Cancer Studyd Group of Japan reported no cases in a survey of over 12,000 liver cancers[26]. Why this remarkable geographical variation should be so is not clear. It occurs in a younger age group and in non-cirrhotic livers and it is possible that different, and as yet unidentified, carcinogenic factors may be involved.

### SCREENING

As with many cancers it has been suggested that early diagnosis offers the best chance of cure. Evidence for this proposal has now emerged from the reports of the Liver Cancer Study Group of Japan[26,38–40]. They suggest that patients with chronic liver disease, particularly cirrhosis, should be followed at regular intervals using ultrasound(US) and measurement of alpha-fetoprotein.

Alpha-fetoprotein is a useful aid in the diagnosis of and screening for hepatocellular carcinoma. Some authors have stated that Western patients with HCC are less likely to have elevated AFP levels than those in the East[41] but intercomparison of such data is difficult. Raised AFP levels are commonly found in chronic hepatitis and cirrhosis and there is no worldwide agreement on what levels should be regarded as significant. In addition detection of tumours at an earlier stage will result in fewer being AFP positive at the time of presentation[42]. Indeed the Liver Cancer Study Group of Japan have found that the incidence of AFP positive patients has decreased from 70% to 50% in successive surveys[26] and they attribute this to the earlier detection of tumours due to screening.

Okuda has recommended that follow up with AFP and ultrasound should be performed every 2–4 months depending on the degree of risk[43]. This policy, which was first applied in the East, has enabled the detection of HCC's at an early stage when partial hepatectomy or limited resection is still possible[18,19,22,26,38–40]. The success of these screening programs should encourage a similar approach in high risk patients in the West.

### SURGICAL TREATMENT

It is important to define indications and contraindications for resection of HCC in patients with liver cirrhosis. Assuming the tumour is anatomically resectable then the level of albumin, bilirubin, prothrombin time and the general nutritional state of the patient are the most important factors. Using Child's classification[44], patients in Child A and B are good surgical candidates. However, perioperative morbidity and mortality also depends on the volume of the operative blood loss and the extent of resection performed. In Japan the value of the indocyanine green (ICG) retention test has been studied[45] as a possible indicator of functional liver cell mass. ICG is removed from the circulation by the liver and its rate of removal therefore reflects liver cell function[46]. The test is, however, time consuming and relatively expensive and the results are sometimes difficult to interpret, especially in the over weight patient. For these reasons it is not commonly used in the West. Perhaps more research is needed into ways of assessing functional liver cell mass in cirrhotic patients where resection is contemplated.

During the last decade, liver surgery has become both safer and more widely practised throughout the world due to technical advances and improved anaesthesiology and perioperative care[47]. In the West there was much initial resistance to the concept of resection in the cirrhotic liver due to problems with intra- and postoperative bleeding and postoperative hepatic failure. In eastern countries, however, a large number of patients with HCC were treated surgically even in the 1970's and the Eastern experience of resection for HCC is vast compared with that of the West. Balasegaram *et al.*[48] carried out 288 resections, of which 99 were for HCC in Kuala Lumpur with a 14.1% of mortality rate in the HCC cases. They stressed the importance of limited resection in the case of severe cirrhosis. Liver resection for HCC is now increasing in western countries where liver surgery has largely been developed on the basis of secondary liver cancer[49] and a gradual increase in the number of HCC cases undergoing hepatectomy in the cirrhotic liver has been reported in western Europe[50]. In the USA, Iwatsuki *et al.*[51] have reported their enormous experience with 411 hepatic resections, in which they showed a gradual increase of HCC resections with a mortality of 7.3%. Most of these were major hepatic resections involving more than two segments (88%) and most of the fatal cases were complicated by cirrhosis. They also pointed out that recurrence dominated the actuarial survival rate after resection for primary malignancy, and their survival figures were 68.5% and 31.9% at 1 and 5 years, respectively.

Follow-up studies after hepatectomy for HCC from some institutions have shown remarkably high intrahepatic recurrence rates of 50% or more, particularly within one to two years of operation[52–54]. This has led some authors to recommend more radical surgery. It is difficult, however, to distinguish recurrence due to nonexcised cancer tissue, unrecognized multifocal primary tumours or new primary tumours. Chen *et al.*[55] examined five recurrent hepatocellular carcinomas in HBsAg positive patients and found that in three cases the clonality of the second cancer differed. Tumour thrombus of the portal vein freuently occurs with tumours 3–5 cm in diameter[38] and resection with limited margins might result in tumour dispersal due to manipulation[56]. Yoshida *et al.*[57] examined the surgical resection margin in relation to recurrence of HCC following resection and suggested that a resection margin of 10mm is inadequate to achieve curability when the tumour size exceeds 4 cm. The problem should be addressed by a prospective trial with intraoperative ultrasonography to ensure second tumours are not left behind and to assess the size of resection margins[58]. Only when such information is available may it be possible to meaningfully study differences in recurrences rates between different centres.

Tumours which are unresectable due either to poor liver function or anatomical reasons have been treated by liver transplantation if extra-hepatic spread has not occurred. Liver transplantation has until now been performed largely in the USA and Western Europe and has not been an option in the Far East and Japan. The published results of liver transplantation for HCC have, however, been disappointing[59–63]. Iwatsuki *et al.*[62] reported that two-thirds of the patients developed tumour recurrence and less than a quarter of the patients survived 3 years. However, patients referred for transplantation are usually those whose tumour is not suitable for conventional resection and this will immediately bias the results. Also when transplantation has been carried out for end-stage cirrhosis with incidental malignancy very much better results have been reported[59,62]. No controlled trial of liver resection versus liver transplantation as a treatment for HCC in the cirrhotic liver has been published and the question of which might be the best treatment remains unanswered.

### CONCLUSION

Hepatocellular carcinoma remains a largely untreated disease worldwide[64] and unfortunately the countries with the highest incidence often have the poorest resources to deal with it. There is currently no firm evidence to suggest that, except for the fibrolamellar variant, the fundamental nature of HCC differs between high-risk and low-risk countries, although there is a difference in the nature of the associated cirrhosis. The true role of the HBV, and possibly other hepatitis viruses[12] as initiators has not yet been fully determined and the future of this exciting aspect of research lies with the molecular biologists. In areas where HBV is endemic an active childhood immunisation policy may be useful[65,66] and several such programs have already begun[66].

The best hope of cure, at present, is surgical removal after detection of the tumour at an early stage. In the future liver transplantation may play an increasing role in developed countries, especially for a small tumour in a severely cirrhotic liver. In Great Britain and Europe more patients should be referred for assessment to specialist centres and patients with chronic liver disease should be considered for regular screening. Finally, in order to learn more about this tumour and study what differences there may be in its behaviour worldwide, we need carefully constructed surveys and screening programs of which those in Taiwan and Japan are currently the best examples.

### *References*

1. Rustgi, V.K. (1988) Epidemiology of hepatocellular carcinoma, *Ann. Intern. Med.*, **108**, 390–401
2. London, W.T. (1981) Primary hepatocellular carcinoma — etiology, pathogenesis, and prevention. *Hum. Pathol.*, **12**, 1085–97
3. Sandler, D.P., Sandler, R.S. and Horney, L.F.F. (1983) Primary liver cancer mortality in the United States. *J. Chronic. Dis.*, **36**, 227–36
4. Munoz, N. and Bosch, X. (1987) Epidemiology of hepatocellular carcinoma. In Okuda, Ishak (Eds.) *Neoplasms of the liver*, pp.3-19. Springer-Verlag
5. Lieberman, H.M., Tur-Kaspa,R. and Shafritz,D.A. (1987) Hepatitis B virus infection and hepatocellular carcinoma. In: Okuda, Ishak (Eds.) *Neoplasms of the liver*, pp.21–33. Springer-Verlag
6. Beasley, R.P., Hwang, L.Y., Lin, C.C. and Chien, C.S. (1981) Hepatocellular carcinoma and hepatitis B virus — a prospective study of 22,707 men in Taiwan. *Lancet*, **2**, 1129–33
7. Sherlock, S. (1989) Hepatic tumors. In: *Diseases of the liver and biliary system*, Eighth Edition, pp.584–617. Blackwell Scientific Publications
8. Bosch, F.X. and Munoz, N. (1988) Epidemiology of hepatocellular carcinoma In: Bannasch P, Keppler D, Weber G, eds. *Falk symposium 51. Liver Cell Cancer*, pp.3–14. Kluwer Academic Publishers
9. Dusheiko, G.M. (1988) Is there a direct genetic role for hepatitis B virus in oncogenesis? In: Bannasch P, Keppler D, Weber G, eds. *Falk symposium 51. Liver Cell Cancer*, pp.135–138. Kluwer Academic Publishers
10. Summers, J., O'Connell, A., Maupas, P., Goudeay, A., Coursaget, P. and Drucker, J. (1978) Hepatitis B virus DNA in primary hepatocellular carcinoma tissue. *J.Med.Virol.*, **2**(3): 207–214
11. Brechot, C., Wain-Hobson, S., Pourcel, C., Dejean, A., Hadchouel M., Scott, J. and Tiollais, P. (1983) Hepatitis B virus DNA and human hepatocellular carcinoma. Progress in clinical and biological research 132D: 495–504
12. Kew, M.C., Houghton, M., Choo, Q.L. and Kuo, G. (1990) Hepatitis C virus antibodies in southern African blacks with hepatocellular carcinoma. *Lancet*, **335**, 873–874
13. Smith, L.H, Jr. (1979) Cancer in cirrhotic liver. In: *Cirrhosis, Major problems in internal medicine Galambos Vol.XVII* pp.363–8. W.B. Saunders Company
14. Lee, C.S.,Sung, J.L., Hwang, L.Y., Sheu, J.C., Chen, D.S.,Lin, T.Y. and Beasley, R.P. (1986) Surgical treatment of 109 patients with symptomatic and asymptomatic hepatocellular carcinoma. *Surgery*, **99**(4): 481–90
15. Tang, Z.Y., Yu, Y.Q., Zhou, X.D., Ma, Z.C., Yang, R., Lu, J.Z., Lin, Z.Y. and Yang, B.H. (1989) Surgery of small hepatocellular carcinoma. Analysis of 144 cases. *Cancer*, **64**, 536–41
16. Nagasue, N., Yukaya, H., Ogawa, Y., Sasaki, Y., Chang, Y.C. and Niimi, K. (1986) Clinical experience with 118 hepatic resections for hepatocellular carcinoma. *Surgery*, **99**(6): 694–701
17. Kew, M.C. and Popper, H. (1984) Relationship between hepatocellular carcinoma and cirrhosis. *Sem. Liver Dis.* **4**, 136–46
18. Nagao, T., Goto, S., Kawano, N., Inoue, S., Mitzuta, T., Morioka, Y. and Omori, Y. (1987) Hepatic resection for hepatocellular carcinoma. Clinical features and long-term prognosis. *Ann. Surg.*, **205**(1), 33–40
19. Kew, M.C. (1988) Role of cirrhosis in hepatocarcinogenesis In: Bannasch P, Keppler D, Weber G, eds. *Falk symposium 51. Liver Cell Cancer*. pp.37–45. Kluwer Academic Publishers
20. Johnson, P.J., Krasner, N., Portmann, B., Eddleston, A.L.W.F. and Williams R. (1978) Hepatocellular carcinoma in Great Britain: influence of age, sex, HBsAg status and aetiology of underlying cirrhosis. *Gut*, **19**, 1022–1026
21. Linsell, C.A. and Peers, F.G. (1977) Aflatoxin and liver cell cancer. *Trans. R. Soc. Trop. Med.*, **71**, 471

22. Simpson, I.W. (1982) Membranous obstruction of the inferior vena cava and hepatocellular carcinoma of South Africa. *Gastroenterology,* **82**, 171
23. Nakashima, T., Okuda, K., Kojiro, M., Sakamoto, K., Kubo, Y. and Shimokawa Y. (1975) Primary liver cancer coincident with Schistosomiasis japonica. *Cancer,* **36**, 1483
24. Purtilo, D.T. (1976) Clonorchiasis and hepatic neoplasms. *Tropical and Geographical Medicine,* **28**, 21
25. Okuda, K., Peters, R.L. and Simson, I.W. (1984) Gross anatomic features of hepatocellular carcinoma from three disperate geographic areas. *Cancer,***54**, 2165–73
26. The Liver Cancer Study Group of Japan. (1990) Primary Liver Cancer in Japan. *Ann. Surg.* **211**(3), 277–287
27. Hsu, H.C., Sheu, J.C.,Lin, Y.H. Chen, D.S., Lee, C.S., Hwang, L.Y. and Beasley, R.P. (1985) Prognostic histologic features of resected small hepatocellular carcinoma in Taiwan. *Cancer,* **56**, 672–680
28. Okuda, K., Musha, M., Nakajima, Y., Kubo, Y., Shimokawa, Y., Nagasaki, Y., Sawa, Y., Jinnouchi, S., Kaneko, T., Obata, H., Hismitsu, T., Motoike, Y., Okazaki, N., Kojiro, M., Sakamoto, K. and Nakashima, T. (1977) Clinicopathologic features of encapsulated hepatocellular carcinoma. *Cancer,* **40**, 1240–1245
29. Nakashima, T., Okuda, K., Kojiro, M., Jimi, A., Yamaguchi, R., Sakamoto, K. and Ikari, T. (1983) Pathology of hepatocellular carcinoma in Japan. 232 consecutive cases autopsied in ten years. *Cancer,* **51**, 863–77
30. Nagorney, D.M., van Heerden, J.A., Ilstrup, D.M. and Adson, M.A. (1989) Primary hepatic malignancy: Surgical management and determinants of survival. *Surgery,* **106**, 740–9
31. Kemeny, F., Vadrot, J., Wu, A., Smadja, C., Meakins, J.L. and Franco, D. (1989) Morphological and histological features of resected hepatocellular carcinoma in cirrhotic patients in the west. *Hepatology,* **9**(2) 253–7
32. Ezaki, T., Kanematsu, T., Okamura, T., Sonoda, T. and Sugimachi, K. (1988) DNA analysis of hepatocellular carcinoma and clinicopathologic implications. *Cancer,* **61**, 106–9
33. Sheu, J.C., Sung, J.L., Chen, D.S., Yang, P.M., Lai, M.Y., Lee, C.S., Hsu, H.C., Chuang, C.N., Yang, P.C., Wang, T.H., Lin, J.T., Lee, C.Z. (1985) Growth rate of asymptomatic hepatocellular carcinoma and its clinical implications. *Gastroenterology,* **89**, 259–66
34. Ebara, M., Ohto, M., Shinagawa, T., Sugiura, N., Kimura, K., Matsutani, S., Morita, M., Saisho, H., Tsuchiya, Y. and Okuda K. (1986) Natural history of minute hepatocellular carcinoma smaller than three centimeters complicating cirrhosis. *Gastroenterology,* **90**, 289--98
35. Nakashima, T., Kojiro, M. (1987) Gross features and gross classification of hepatocellular carcinoma. In: *Hepatocellular carcinoma-An atlas of its pathology,* pp.3–8. Springer-Verlag
36. Kohno, H., Nagasue, N., Taniura, H., Nakamura, T. and Nagaoka, S. (1988) Fibrolamellar carcinoma of the liver. A case report from Japan and a review of the literature. *HPB Surgery,* **1**, 77–80
37. Paradinas, F.J., Melia, W.M., Wilkinson, M.L., Portmann, B., Johnson, P.J., Murray-Lyon, I.M. and Williams, R. (1982) High serum vitamin $B_{12}$ binding capacity as a marker of the fibrolamellar variant of hepatocellular cancer. *Br. Med. J.,* **285**, 840–842
38. Okuda, K. (1980) The Liver Cancer Study group of Japan. Primary liver cancer. *Cancer,* **45**, 2663–9
39. The Liver Cancer Study Group of Japan (1984) Primary liver cancer in Japan. *Cancer,* **54**, 1747–55
40. The Liver Cancer Study Group of Japan (1987) Primary liver cancer in Japan — Sixth report. *Cancer,* **60**, 1400–11
41. Geography of primary liver cancer. (1990) Leading Article. *Br. Med. J.,* **6**, 381–382
42. Sawabu, N. and Hattori, N. (1987) Serological tumour markers in hepatocellular carcinoma. In: Okuda K. Ishak KG, eds. *Neoplasms of the liver,* pp.227–249. Springer-Verlag
43. Okuda, K. (1988) Early recognition of liver cell tumours. In: Bannasch P, Keppler D, Weber G, eds. *Falk symposium 51. Liver Cell Cancer,* pp.439–453. Kluwer Academic Publishers
44. Child, C.G. and Turcotte, J.G. (1964) Surgery in portal hypertension. In: Child CG, ed. *Major problems in clinical surgery: The liver and portal hypertention,* pp.1–85. Philadelphia: W.B. Saunders
45. Ezaki, T., Yukaya, H. and Ogawa, Y. (1987) Evaluation of hepatic resection for hepatocellular carcinoma in the elderly. *Br.J.Surg.,* **74**, 471–3
46. Sherlock, S. (1989) Assessment of liver function. In: *Diseases of the liver and biliary system,* pp.19–35. Eighth edition. Blackwell Scientific Publications
47. Matsumata, T., Kanematsu, T., Shirabe, K., Sonoda, T., Furuta, T. and Sugimachi, K. (1990)

Decreased morbidity and mortality rates in surgical patients with hepatocellular carcinoma. *Br.J.Surg.*, **77**, 677–680
48. Balasegaram, M. and Joishy, S.K. (1981) Hepatic resection. Pillars of success built on the foundation of 15 years of experience. *Am.J.Surg.*, **141**, 360–65
49. Registry of hepatic metastases (1988) Resection of the liver for colorectal carcinoma metastases: A multi-institutional study of indications for resection, *Surgery*, **103**, 278–87
50. Bismuth, H., Houssin, D., Ornowski, J. and Meriggi, F. (1986) Liver resections in cirrhotic patients: A western experience. *World. J. Surg.*, **10**, 311–7
51. Iwatsuki, S. and Starzl, T.E. (1988) Pesonal experience with 411 hepatic resections. *Ann.Surg.*, **208** (4), 421–34
52. Lin, T.Y., Lee, C.S., Chen, K.M. and Chen, C.C. (1987) Role of surgery in the treatment of primary carcinoma of the liver: a 31-year experience. *Br.J.Surg.*, **74**(9), 839–42
53. Kanematsu, T., Matsumata, T., Takenaka, K., Yoshida, Y., Higashi, H. and Sugimachi, K. (1985) Clinical management of recurrent hepatocellular carcinoma after primary resection. *Br.J.Surg.*, **75**(3), 203–6
54. Nagao, T., Inoue, S., Yoshimi, F., Sodeyama, M., Omori, Y., Mizuta, T., Kawano, N. and Morioka, Y. (1990) Postoperative recurrence of hepatocellular carcinoma. *Ann.Surg.*, **211**(1), 28–33
55. Chen, P.J., Chen, D.S., Lai, M.Y. *et al.* (1985) Clonal origin of recurrent hepatocellular carcinoma. *Gastroenterology*, **96**, 527–529
56. Ezaki, T., Yukaya, H., Ogawa, Y., Chang, Y.C. and Nagasue, N. (1989) Recurrent form of hepatocellular carcinoma after partial hepatic resection. *Hepato-gastroenterol.*, **36**, 164–7
57. Yoshida, Y., Kanematsu, T., Matsumata, T., Takenaka, K. and Sugimachi, K. (1989) Surgical margin and recurrence after resection of hepatocellular carcinoma in patients with cirrhosis. Further evaluation of limited hepatic resection. *Ann.Surg.*, **209**, 297–301
58. Ezaki, T., Stansby, G.P. and Hobbs, K.E.F. (1990) Intraoperative ultrasonographic imaging in liver surgery: A review. *HPB Surgery*, (in press)
59. Iwatsuki, S., Gordon, R.D., Show, B.W. Jr. and Starzl, T.E. (1985) Role of liver transplantation in cancer therapy. *Ann.Surg.*, **202**, 401–7
60. O'Grady, J.G., Polson, R.J., Calne, R.Y. and Williams, R. (1986) Liver transplantation for malignant disease: results in 93 consecutive patients. *Ann.Surg.*, **207**, 373–9
61. Ringe, B., Wittekind, C., Bechstein, W.O., Bunzendahl, H. and Pichlmayr, R. (1989) The role of liver transplantation in hepatobiliary malignancy. A retrospective analysis of 95 patients with particular regard to tumor stage and recurrence. *Ann.Surg.*, **209**, 88–98
62. Iwatsuki, S. and Starzl, T.E. (1987) Liver transplantation in the treatment of liver cancer. In: Okuda, Ishak (Eds.) *Neoplasms of the liver*, pp.397–405. Springer-Verlag
63. Ismail, T., Angrisani, L., Gunson, B.K., Hubscher, S.G., Buckels, J.A.C., Neuberger, J.M., Elias, E. and McMaster, P. (1990) Primary hepatic malignancy: the role of liver transplantation. *Br.J.Surg.* **77**, 983–987
64. Maraj, R., Kew, M.C. and Hyslop, R.J. (1988) Resectability rate of hepatocellular carcinoma in rural Southern Africans. *Br.J.Surg.*, **75**, 335–8
65. Deinhardt, F. and Jilg, W. (1988) Strategies in prevention of hepatocellular carcinoma by vaccination. In: Bannasch, P., Keppler, D., Weber, G., (eds). *Falk symposium 51. Liver Cell Cancer*, pp.509–516. Kluwer Academic Publishers
66. Hall, A.J., Greenwood, B.M. and Whittle, H. (1990) Modern Vaccines — Practice in developing countries. *Lancet*, **335**, 774–7

